# Race and Ethnic Categories: A Brief Review of Global Terms and Nomenclature

**DOI:** 10.7759/cureus.41253

**Published:** 2023-07-01

**Authors:** Catherine Lewis, Philip R Cohen, Devyani Bahl, Elliot M Levine, Waseem Khaliq

**Affiliations:** 1 General Surgery, East Tennessee State University, Johnson City, USA; 2 Dermatology, University of California Davis Medical Center, Sacramento, USA; 3 Oral and Maxillofacial Surgery, NITTE University, Mangalore, IND; 4 Obstetrics and Gynecology, University of Illinois at Chicago, Chicago, USA; 5 Obstetrics and Gynecology, Advocate Illinois Masonic Medical Center, Chicago, USA; 6 Medicine, Johns Hopkins University School of Medicine, Baltimore, USA

**Keywords:** race, native hawaiian, hispanic, ethnicity, caucasian, black, asian, american indian, african american

## Abstract

Terminology regarding descriptors of race and ethnicity have been constantly evolving. Due to differences in terminology, data collection, demographics, and group identity, there are numerous challenges in determining what descriptors are suitable and acceptable to all individuals. The National Institutes of Health (NIH) has defined six racial and ethnic categories that should be used for reporting purposes. This review gives a historical background of the definition of the different racial and ethnic categories. This review also aims to define acceptable categories of race and ethnicity to provide guidelines for reports and best practices.

## Introduction and background

Information regarding race has been systematically collected by the United States (US) Census Bureau since the first census in 1790 [1]. The terminology regarding descriptions of race and ethnicity have not been consistent and has evolved over the years. Differences in terminology, methods of data collection, individual perceptions of group identity, and changing demographics present challenges in determining racial and ethnic categories that are specific and acceptable to all individuals [2]. 

Confusion between race and ethnicity also presents challenges [3,4]; indeed, when searching the medical literature using PubMed, those terms are often combined (e.g., race and ethnicity). Historically, race has been defined based on physical characteristics such as skin, hair, or eye color. Race was defined based upon scientific observations that Europeans had with people from different cultural and political regions. Race was considered a damaging construct that was used to divide different groups of people, making one race superior over another. The definition of race is now widely accepted as being a socially constructed term, without any biological basis [3,5]. Ethnicity is defined as cultural factors such as language, religion, cuisine, ancestry, and nationality that specific communities share. Ethnicity is also considered a social construct that individuals may change as their community and personal dynamics change [3].

In the current manuscript, we aim to describe the history of racial and ethnic categories and to place them in the context of medicine and the language that is used. We also aim to define acceptable categories of race and ethnicity and the impact of their use in the scientific and medical communities.

## Review

Definitions

The Office of Management and Budget issued Statistical Directive 15, “Race and Ethnic Standards for Federal Statistics and Administrative Reporting” in 1978 [1,6]. This policy provided guidelines for categories for the collection of data on race and ethnicity in the United States (US). The categories correspond to the major continents. This policy was revised in 1997 with the following six racial and ethnic categories: American Indian or Alaska Native, Asian, Black, or African American, Hispanic, or Latino, Native Hawaiian or Other Pacific Islander, and White. The National Institutes of Health (NIH) has also defined racial and ethnic categories for reporting purposes (Table 1) [7].

**Table 1:** National Institutes of Health (NIH) racial and ethnic categories.

Racial and ethnic category	Definition
American Indian or Alaska Native	A person having origins in any of the original peoples of North and South America (including Central America), and who maintains tribal affiliation or community attachment
Asian	A person having origins in any of the original peoples of the Far East, Southeast Asia, or the Indian subcontinent, including Cambodia, China, India, Japan, Korea, Malaysia, Pakistan, the Philippine Islands, Thailand, and Vietnam
Black or African American	A person having origins in any of the black racial groups of Africa. Terms such as “Haitian” or “Negro” can be used in addition to “Black or African American.”
Hispanic or Latino	A person of Cuban, Mexican, Puerto Rican, South or Central American, or other Spanish culture or origin, regardless of race. The term “Spanish origin” can be used in addition to “Hispanic or Latino.”
Native Hawaiian or Other Pacific Islander	A person having origins in any of the original peoples of Hawaii, Guam, Samoa, of other Pacific Islands
White	A person having origins in any of the original peoples of Europe, the Middle East, or North Africa.

American Indian

American Indians and Alaska Natives have been the most difficult groups to describe. The NIH defines an American Indian or Alaska Native as “a person having origins in any of the original peoples of North and South America (including Central America), and who maintains tribal affiliation or community attachment [7].” The 1790 Census distinguished “Indians” only for purposes of taxation. The term “American Indian” was first used in the 1960 Census. In 1970, the Census asked for specific Indian tribes [6]. Guidelines vary widely depending on tribal affiliation. Tribes can be federally recognized, state-recognized, or unrecognized, and there is a great amount of self-identity in this group of people [3]. 

Asians

Asian is defined as “a person having origins in any of the original peoples of the Far East, Southeast Asia, or the Indian subcontinent [7].” In the United Kingdom (UK), categories are identified by colloquial terms that specific groups may use to self-identify. Racial and ethnic terms are more interchangeable. The UK categorized those of Asian descent as Asian, Asian British, including Indian, Pakistani, Bangladeshi, or Chinese [8]. Asian Americans are a race defined by at least thirty separate ethnic groups and many more cultural groups in the US census [9]. Racial experiences are one factor that has influenced the growth of a pan-Asian American identity. Asian American was used to refer to American Asians and to post-1965 arrivals to the US with the intention of permanent settling. Asian Pacific American was used by the government for administrative convenience [10].

Blacks

In 1890, the US Census further classified the population as white, black, mulatto, quadroon, octoroon, Chinese, Japanese, or Indian. Statistical Directive 15 was important because it acknowledged that the US population was made up of more than just black and white people [6]. Black or African American is defined as “a person having origins in any of the black racial groups of Africa [7].” However, Northern Africans from Algeria and Morocco are excluded and are classified as “Whites [11].” The prevailing belief that “one drop” of African blood defines an African American is still present in the US [12].

The first US Census classified the population as free white males, free white females, other persons, and slaves. Under the apartheid government of South Africa, racial groups were defined as “colored, white, and native.” White/European, African, colored, and Asian are still widely used in healthcare settings in South Africa. PubMed/MEDLINE previously defined and cataloged racial categories as “Caucasoid, Mongoloid, Negroid, and Australoid” until 2003 [12]. In Europe, certain minority groups are considered easier to define, such as Afro/Black Europeans. In the UK, Black people are categorized as Black, African, Caribbean, or Black British [8].

Hispanic and Latino

The Hispanic origin concept began in 1970 in the US with the self-identification approach. The US Census identified Spanish-Hispanic populations based on birthplace, mother tongue, and Spanish surname prior to 1970 [1]. Hispanic or Latino is defined as “a person of Cuban, Mexican, Puerto Rican, South or Central American, or other Spanish culture or origin, regardless of race [7].” Consistency in responses to the Hispanic heritage question has changed over time and varies depending on location. For example, people of Portuguese descent in Massachusetts and Rhode Island often identify as Hispanic [1]. In 1960, Puerto Ricans, Mexicans, and other people of Latin descent were classified as White unless they were specifically “Negro, Indian, or some other race [6].” Women from the island of Puerto Rico identify as White, Black, or trigueña, while those from the US mainland identify as Hispanic/Latina, Hispanic American, or American [13]. “Boricua de Pura Cepa” translates to “purebred Boricua/Puerto Rican, Puerto Rican of pure strain/stump, or Puerto Rico to the bone,” and is used to express pride and self-identification as Puerto Ricans [14].

Native Hawaiian or other Pacific Islander

Native Hawaiian or other Pacific Islander is “a person having origins in any of the original peoples of Hawaii, Guam, Samoa, or other Pacific Islands [7].” This is one of the fastest-growing populations in the US [1]. In Australia, “panethnicity” is an important factor of identity for Pacific Islanders who identify as Fijians, Papua New Guineans, Samoans, Tuvaluans, and Tongans [15]. There are demographic distinctions between Pacific Islanders in the US as compared to those in Australia and New Zealand [16]. These differences may contribute to the cultural differences that may define ethnic descriptions.

Whites

A White person is defined as “a person having origins in any of the original peoples of Europe, the Middle East, or North Africa [7].” Other descriptors include Caucasian, Anglo, or the nationalities of the person [17]. The categories in the UK combine national, ethnic, and geographic headings. White, including Gypsy or Irish travelers, is a category. In some European countries, White as a category is a difficult concept as some ethnic minorities would be classified as White, thereby undermining claims for special rights [8].

Recommended terminology

Negro

The concept of ethnicity is slowly replacing the concept of race. However, there are many concepts that overlap between race and ethnicity. Despite the overlap, there are certain labels that need to be phased out, replaced, or both. “Negro,” referring to the color black in Spanish, was used by Europeans as a shortened version of the term “Negroid” to describe people from sub-Saharan African. Today, the term is only acceptable in historical contexts or in the name of organizations, such as the United Negro College Fund (UNCF). It is considered derogatory and inappropriate for use [11,18].

Black

The term Black has been used to qualify all non-White minorities. Although this term can cover a wide range of ethnic backgrounds, it can also be seen as offensive. It is also unreliable and too simple to encompass mixed ethnicities and races. “African” is the preferred prefix to describe more specific categories, such as African American or African Caribbean [11,18].

Asian

The term Asian is used slightly differently in the US as compared to the UK. Similar to the African prefix, the term Asian should be used to describe more specific categories [19]. Detailed descriptors such as “Chinese American” or “Pakistani” should be used instead of grouping into a single Asian group. This should also be standard for Pacific Islanders [20].

Caucasian

Johann Friedrich Blumenbach was a German anthropologist who was instrumental in developing a classification of humankind. He is also referred to as the inventor of the “Caucasian race.” In the 1795 version of his thesis, he defined five generic categories: Caucasian, Mongolian, Ethiopian, American, and Malayan. He described “White Europeans” as “Caucasians [21].” Despite previously recommending the abandonment of the term Caucasian [17], it remains popular and acceptable in scientific writings and common language [11]. However, others recommend the use of “White” when referring to race [20].

Social-not scientific-constructs

Medical literature has historically used race and ethnicity to address disparities and inequities in health. However, it is crucial to recognize that race and ethnicity are not inherently linked to biology or genetics. Instead, race and ethnicity are social constructs shaped by historical and cultural factors. There is significant variation in how individuals and communities self-identify to race and ethnicity and how healthcare providers and researchers define and report these categories. Therefore, standardization of terminology is essential for promoting clarity, accuracy, and inclusivity in medical literature and reporting while keeping in mind associated sensitivities and controversies related to race and ethnicity [8,20,22,23]. Although it may be common in the healthcare setting for a patient to be asked about their self-identification, the limited diagnostic impact it has should be recognized.

The statistical incidence of a disease or condition in a particular ethnic population may be helpful in producing a differential diagnosis. Because of the constant changes in ethnic self-identification, it is crucial to not inappropriately exclude other diagnostic possibilities. This is especially important since healthcare disparities have been shown to result from these characterizations and focus on scientific constructs.

Guidelines for reporting

Several guidelines are available to help report accurate and precise use of race and ethnicity, reflecting the appropriate and equitable choice of words in the medical literature [8,20,22,23]. Despite the guidelines, there is a lack of consensus about a unified definition of race and ethnicity, either due to inconsistency in using culturally and linguistically appropriate methods for collecting and reporting data or difficulty in ascertaining this information using standardized terminology. Additionally, a lack of an individual’s understanding of why and how this collected information is utilized poses a challenge due to changes in self-reported identities over time, especially in multiracial and multiethnic groups. Efforts to standardize the reporting of race and ethnicity data are ongoing. While progress has been made, there are still challenges associated with reporting standards, both within and across continents [23].

Expression of an individual’s identity

Race and ethnicity are frequently defined in terms of cultural affiliation, physical appearance, and self-reported geography. These traditional racial or ethnic categories might not be able to fully capture the complexity of an individual’s identity, especially in the case of migrants that come from diverse cultural and linguistic backgrounds. Thus, there is a growing recognition of the limitations of considering race or ethnicity as an absolute category due to cross-continent migration, increased multiculturalism, and heightened awareness of complex racial and ethnic identities. One approach to address these issues is to expand the category of “other,” where people self-identify as having multiracial or multiethnic heritage [23]. Although race and ethnicity are complex and multifaced concepts, data is more reliable when self-reported by an individual. Even self-reported identities can change over time depending on the individual’s affiliation with their community, perceptions of acceptance, advantages or disadvantages, and safety concerns [24].

Genetic ancestry tests

The terminology and understanding of race and ethnicity are constantly evolving, and the availability of genetic ancestry tests has added a new layer to this discussion. These tests can provide a deeper understanding of someone’s genetic ancestry and heritage, which may lead to a re-evaluation of their racial or ethnic identity.

It is important to note that genetic ancestry tests have limitations. They may only provide a partial view of an individual’s heritage and may not always align with their self-identification or cultural affiliation. Moreover, using genetic information to define racial or ethnic identity poses challenges and controversies, and it is essential to approach these discussions with sensitivity and respect [25,26]. Significant challenges remain regarding how this information will be collected and utilized without perpetuating stereotyping for research purposes. Ultimately, an individual’s racial or ethnic identity should be self-defined, and it’s up to each individual to decide how they want to identify and be recognized.

Establishing best practices

In the near future, as the use of ancestral pedigree and genetic ancestry tests become widely available, it will be essential to establish ethical guidelines and best practices for collecting and using genetic ancestry information in research. This will require dialogue and collaboration between researchers, geneticists, social scientists, and community stakeholders to ensure that the collection and use of genetic ancestry information is done responsibly and respectfully [27]. The goal should be to use such information to better understand the association of race and ethnicity with healthcare disparities and outcomes. A global consensus is required to standardize race and ethnicity reporting utilizing ancestry when available. This can likely be achieved by a consistent and careful choice of words to ascertain self-reported race and ethnicity in a manner that maximizes the potential benefit to the study population and avoid stigmatization or perpetuating racism.

## Conclusions

The definition of race and ethnicity is an ever-changing concept. Despite the six racial categories that have been suggested for use by the National Institutes of Health (NIH), there is wide variation in self-identity due to variations in cultural beliefs, geography, language, religion, and even physical appearance. As such, race and ethnicity are complex phenomena without straightforward definitions.

Collecting race and ethnicity information can provide important insights into population health, including disease prevalence, health behaviors, outcomes, and disease management. As we move towards personalized and precision medicine, this information will continue to provide valuable and critical information to develop targeted interventions to address disparities in health outcomes between racial and ethnic groups.
